# *Sirt*ainties in pancreatic cancer?

**DOI:** 10.18632/oncoscience.28

**Published:** 2014-04-03

**Authors:** Ilse Rooman

**Affiliations:** The Garvan Institute of Medical Research / The Kinghorn Cancer Centre, Darlinghurst, Australia

Sirtuin 1 (Sirt1) is a gene with diverse and complex roles in metabolic regulation, tumorigenesis and longevity, the latter being highly debated. Sirt1 is one of seven sirtuins that are NAD^+^ dependent and that, depending on the family member, modify histone and non-histone proteins by deacetylation and/or mono-ADP-ribosylation. Excellent reviews detail the long list of Sirt1 targets for deacetylation and capture its functions in homeostasis and disease [1, 2].

Recently, we reported a novel role for Sirt1 in pancreatic disease [3]. Until then, Sirt1 studies in the pancreas were confined to insulin producing endocrine cells. We investigated Sirt1 in the acinar cells of the pancreas that secrete digestive enzymes, and are prominent players in exocrine pancreatic disease. Acinar cells undergo ductal metaplasia when under stress, exemplified by the events in chronic pancreatitis where this can be a precursor to pancreatic ductal adenocarcinoma (PDAC) [4]. PDAC is the most prevalent form of pancreatic cancer and a lethal tumour that limits the median patient survival to less than 6 months after diagnosis. An increased understanding of pancreatic biology, both in pancreatitis and pancreatic cancer is warranted.

**Figure 1:**
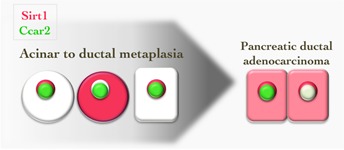
Graphic illustration of the expression of Sirt1 and its endogenous inhibitor Cacr2 in pancreatic acinar to ductal metaplasia and in progressed pancreatic ductal adenocarcinoma Altered Sirt1 interactions with down stream target proteins and altered Sirt1 activity are likely to ensue from the shift in Sirt1 intracellular localization as well from changes in Ccar2 expression levels.

Our work described Sirt1's expression related to that of Ccar2 (Cell cycle and apoptosis regulator 2, also named Deleted in breast cancer 1 or KIAA1967), a protein that directly inhibits the deacetylation activity of Sirt1 (Figure 1). While in the unstressed pancreas both proteins colocalize in the nucleus, in acinar to ductal metaplasia Sirt1 temporary relocates to the cytoplasm while Ccar2 remains nuclear. This allows altered interaction of Sirt1 with its target genes, here, Pancreatic transcription factor 1a and beta-Catenin that critically regulate acinar cell differentiation and ductal metaplasia [4], and possibly other proteins. While SIRT1 is variably expressed in most of the human PDAC, CCAR2 is downregulated in a subset. These observations suggest that altered Sirt1 activity through changed intracellular localization and interaction with Ccar2 is important in different stages of pancreatic cancer development, revealing potential therapeutic opportunities. Indeed, Sirt1 inhibitors have generated excitement as therapeutic agents [1]. One of them, nicotinamide, attenuates the incidence of hepatocellular carcinoma without any evidence of toxic effects for the mice and is being tested in a clinical trial as a preventative agent for skin cancer. Our study showed that addition of nicotinamide partially repressed pancreatic acinar to ductal metaplasia. Other Sirt1 inhibitors, like Tenovin-6, showed anti-cancer effects in cell lines and mouse models, through activation of p53 and other mechanisms [1]. Another Sirt1 inhibitor (EX-527) is in clinical trial, albeit for Huntington's Disease and not (yet) for cancer [1]. In our study, several PDAC cell lines were sensitive to Sirt1 inhibition, an observation that is extended by others who showed that Sirt1 inhibitors increase chemosensitivity of PDAC cells to the clinically applied drug gemcitabine [5, 6].

The latter data strongly suggest an oncogenic role of Sirt1 in late stage PDAC. Several Sirt1 mouse models are available to shed light on its potential contribution to PDAC development. The mouse models are based on impairment of Sirt1's catalytic subunit through deletion of a critical exon or point mutation, and have been used in a whole body as well as in a tissue-restricted context [1, 2]. Complementary, a Sirt1 transgenic mouse has been widely studied [1, 2]. These mouse models, however, have illustrated the complexity of Sirt1 and have not uncommonly generated data that are hard to reconcile. One such example is the role in the intestine where both Sirt1 inactivation [7] and Sirt1 overexpression [1, 2] in the APC Min model of colorectal cancer led to reduced tumor development. For PDAC studies, several important aspects may be missing in the genetically engineered mice, such as the contextual effects of Sirt1 being shuttled in and out of the nucleus impacting on Sirt1's accessibility to different targets for deacetylation. Another factor is the interaction with endogenous regulators such as Ccar2, as well as possible compensatory effects by the closely related Sirt2, mostly located in the cytoplasm and shown to have a tumor suppressive role [1]. Nevertheless, an integrative study using complementary models will provide new insights.

The *in vivo* part of our study to date was limited to experimental acute pancreatitis by injections of the cholecystokinine analogue caerulein. Our paper demonstrates that the caerulein treated pancreas with Sirt1 inactivation has more pronounced acinar to ductal metaplasia and after a week shows a reduced size compared to controls. The latter could be easily explained by Sirt1's well known inactivating role on stress-induced p53 mediated apoptosis and proliferation block [8]. We attribute the persistence of acinar to ductal metaplasia to the depletion of nuclear Sirt1, with our results suggesting that lack of nuclear Sirt1 and increase in cytoplasmic Sirt1 both independently contribute to acinar to ductal metaplasia.

In no doubt, this study warrants further investigation of the role of Sirt1 in pancreatic cancer development as well as of the chemotherapeutic efficacy of Sirt1 inhibitors in preclinical models of human PDAC, guided by the expression of Sirt1 and Ccar2. Not only may a unique opportunity arise from the use of Sirt1 inhibitors, our studies also caution the proposed use of Sirt1 activators as anti-ageing therapy as long as uncertainties persist on the role of a pleiotrophic protein such as Sirt1.
